# Supplementary material for: Rationale and validation of a novel mobile application probing motor inhibition: Proof of concept of CALM-IT
Source: PLoS One. 2021 Jun 4;16(6):e0252245. doi: 10.1371/journal.pone.0252245 (PMC8177631; doi:10.1371/journal.pone.0252245)
Supplement: S1 File — Description of methods and results of a confirmatory factor analysis of the 18 items measuring ADHD symptoms from the Conners Comprehensive Behavior Ratings Scale. (PDF) [file pone.0252245.s001.pdf]

Rationale and validation of a novel mobile application probing motor inhibition: Proof of concept of CALM-IT

Elise M. Cardinale, Reut Naim, Simone P. Haller, Ramaris German, Christian Zapp, Jessica Bezek, David Jangraw, & Melissa A. Brotman

Supporting Information

Confirmatory Factor Analysis of Conners Comprehensive Behavior Ratings Scale ADHD Items.

In a large sample of clinical youth (n=788, Age M(SD)=12.49(2.65), 50.25% Male) we examined factor loadings of the 18 items included in the Conners Comprehensive Behavior Ratings Scale (CBRS) DSM-IV ADHD Total Subscale. Critically, this sample is comprised of the same patient population proposed in the current project, thus responses to the CBRS in this larger sample can be generalized to the sample recruited for the proposed project. Using confirmatory factor analysis, we tested a model with all 18 items loading onto a single latent factor. Standardized factor loadings were examined for each of the 18 items to select the six items with the highest factor loadings (displayed in table below). Factor loadings for these six items were all large and ranged from 0.829-0.882.

#	Text	Loading
79.	Easily distracted by extraneous stimuli	0.882
41.	Fails to give close attention to details or makes careless mistakes in schoolwork, work, or other activities	0.853
30.	Has difficulty organizing tasks and activities	0.844
50.	Forgetful in daily activities	0.843
29.	Does not follow through on instructions and fails to finish schoolwork, chores or duties in the workplace	0.833

20.	Does not seem to listen to what is being said to him/her	0.829
9.	Avoids, expresses reluctance about, or has difficulties engaging in tasks that require	0.810
10.	Has difficulty sustaining attention in tasks or play activities	0.789
71.	Loses things necessary for tasks or activities (e.g., school assignments, pencils, books, tools or toys)	0.771
49.	Interrupts or intrudes on others (e.g., butts into others' conversations or games)	0.761
42.	Has difficulty waiting in lines or awaiting turn in games or group situations	0.743
55.	Fidgets with hands or feet or squirms in seat	0.733
80.	Blurts out answers to questions before the questions have been completed	0.711
76.	Leaves seat in classroom or in other situations in which remaining seated is expected	0.698
39.	Talks excessively	0.683
3.	Is always "on the go" or acts as if driven by a motor	0.663
59.	Has difficulty playing or engaging in leisure activities quietly	0.653
23.	Runs about or climbs excessively in situations where it is inappropriate	0.635

Note. # = corresponding item number on the Conners Comprehensive Behavior Ratings Scale. Loading = standardized factor loading for each item on the single latent factor
